# Correlation analysis of maternal condition during pregnancy with head circumference and autism spectrum disorder: A propensity score-matched study

**DOI:** 10.1097/MD.0000000000036104

**Published:** 2024-02-09

**Authors:** Lei Liu, Shichun Zhao

**Affiliations:** a Department of Burns and Plastic Surgery, The Second Hospital, Cheeloo College of Medicine, Shandong University, Jinan, Shandong Province, People’s Republic of China; b Department of Paediatrics, The Second Hospital, Cheeloo College of Medicine, Shandong University, Jinan, Shandong Province, People’s Republic of China.

**Keywords:** ASD, FA, macrocephaly, microcephaly

## Abstract

To determine whether health status during pregnancy is associated with autism spectrum disorder (ASD) and abnormal head circumference (HC) in the offspring. This study included 41 Han children with ASD who visited the Children’s Health Clinic of the Second Hospital of Shandong University between March 2018 and February 2019, and 264 Han children with typical development (TD) who visited the clinic during the same period. Physical measurements were performed on the children. The questionnaire obtained information on maternal risk factors that may be related to the increased risk of ASD and folic acid (FA) supplementation. We designed an observational case–control study using propensity score matching and multivariate logistic regression analysis. The incidence of macrocephaly in the ASD group was 22.0%, significantly higher than that in the TD group (1.8%). The incidence of microcephaly in the ASD group was 17.1% (n = 7), significantly higher than that in the TD group (1.8%). The differences between the comparisons were statistically significant. Maternal FA supplementation during pregnancy was significantly associated with ASD (*P* < .05), with an odds ratio (95% confidence interval of 3.69 (1.76, 7.76)). Also was associated with macrocephaly (*P* < .05), odds ratio (95% confidence interval) were 8.13 (1.63, 40.61) and 4.16 (1.18, 14.60), respectively. The incidence of abnormal HC was higher in the ASD group than that in the TD group. Maternal FA supplementation during pregnancy may be negatively associated with the occurrence of ASD and abnormal HC in the offspring. Further examination of the role of maternal health status in the etiology of ASD is recommended.

## 1. Introduction

Autism spectrum disorder (ASD) is a neuro-developmental disorder that may carry a poor prognosis in children. Its core features are social impairment and stereotypical interests and behaviors. These persistent symptoms create a huge burden on individuals, families, and society. Despite increased prevalence over the past 3 decades, genetic factors alone cannot explain this trend. ASD is associated with a wide range of environmental risk factors.^[1]^ Consequently, identifying the underlying causes and preventive factors are the top priorities for current ASD research.

Maternal health, both before and during pregnancy, has been found to significantly impact fetal growth and brain development. Several studies have suggested that dysregulated epigenetic mechanisms, often resulting from exposure to adverse environmental factors in utero and early in life, may play a role in the etiology of ASD.^[2–4]^

Prenatal folic acid (FA) supplementation is important in preventing tube defects,^[5]^ but its effects on head circumference (HC) and ASD risk remain unclear. Several systematic reviews and meta-analyses have shown that prenatal FA use of FA reduces the risk of ASD in children.^[6–9]^ Other studies have not found a significant inverse relationship.^[10–12]^

Abnormal fetal head growth has been proposed as an early biomarker of ASD.^[13]^ However, conclusions regarding HC in children with ASD remain limited, inconsistent, and controversial. A meta-analysis revealed that patients with ASD had significantly larger HC than controls, with 15.7% of patients with ASD exhibiting macrocephaly.^[14]^ Amaral et al^[15]^ suggested that megalencephaly may be a specific phenotype of ASD. Conversely, a national cohort study in Denmark reported no association between microcephaly or macrocephaly, and ASD.^[16]^ Furthermore, the study by Dinstein et al^[17]^ demonstrated no significant correlation between ASD scores at diagnosis and HC during the first 2 years of life.

Currently, only a few studies have investigated maternal FA supplementation during pregnancy and offspring HC. A systematic review conducted in 2021 indicated that maternal plasma folate deficiency in the first trimester is associated with reduced fetal brain volume.^[18]^ Additionally, a population-based prospective cohort study showed a modest independent association between maternal folate concentration in the first trimester and fetal head growth.^[19]^

The primary objective of this study was to investigate the relationship between maternal health status, FA supplement use during pregnancy, HC, and the risk of offspring developing ASD in the offspring. We pursued 2 goals. First, we compared the incidence of abnormal HC between ASD and typical development (TD) groups. Second, we examined the health status and FA supplement use of pregnant women and their offspring with ASD. Our aim was to determine whether maternal FA supplementation was associated with HC in the offspring.

## 2. Materials and methods

### 2.1. Study design and participants

This case–control study was conducted at the Children’s Health Clinic of the Second Hospital of Shandong University, China, between March 2018 and February 2019. Prior to the start of the study, informed consent forms were signed by the caregivers of all participants. Moreover, The Ethics Committee of the Second Hospital of Shandong University officially approved this study. (KYLL-2016(LW)-0020).

#### 2.1.1. ASD group.

This study consisted of 41 Han children with ASD, comprising 30 boys and 11 girls aged 3 to 7 years. The inclusion criteria for participant selection were as follows: (1) compliance with the diagnostic criteria for ASD in the 5th edition of the Diagnostic and Statistical Manual of Mental Disorders, and (2) negative findings on brain magnetic resonance imaging and electroencephalography tests.

The exclusion criteria were as follows: (1) other forms of pervasive developmental disorders, (2) motor developmental delay, or (3) history of underlying or congenital diseases.

#### 2.1.2. TD group.

In the TD group, a total of 264 Han children with TD were recruited, consisting of 149 boys and 115 girls, aged 3 to 7 years. The inclusion criteria for participant selection were as follows: (1) absence of any delay in neuromotor development at any stage and (2) no family history of ASD.

The exclusion criteria were as follows: (1) any form of universal developmental disorder, (2) delayed motor development, and (3) a history of underlying or congenital diseases.

### 2.2. Data collection

Maternal risk factors associated with an increased risk of ASD were assessed through questionnaires administered to mothers. The questionnaires collected information regarding the mother’s age at the time of delivery, educational background, parental history of autoimmune diseases, gestational age, mode of delivery, and pregnancy complications, such as infection, anemia, abnormal thyroid function, hypertension, and hyperglycemia. Additionally, data on the children’s age, sex, and anthropometric measurements were obtained. Furthermore, the mothers were asked to report the use of FA supplements during pregnancy.

To minimize error bias, anthropometric data were measured 3 times and the average value was recorded. The final data is reported with an accuracy of 0.01 (cm, kg).

### 2.3. Statistical analysis

According to the propensity score matching (PSM) analysis plan, with grouping variables (1 = children with ASD, 0 = normal children) as dependent variables and children’s gender and age as matching variables, SPSS 22.0, which automatically uses logistic regression to fit the independent and dependent variables, was used to calculate the propensity score of each sample. The nearest neighbor matching method was used to match the ASD and TD groups at a ratio of 1:1. Set the caliper value to 0.02. After matching was completed, the successfully matched normal child group was removed. This process was continued until it could not be matched completely. A total of 1:4 ASD and TD groups were also matched.

Normality tests were conducted on continuous data including age, height, weight, and HC. Data conforming to a normal distribution were presented as means and standard deviations, while non-normally distributed data are represented by medians and quartile ranges. Between-group comparisons were performed using the t-test for data with a normal distribution and the Mann–Whitney U rank-sum test for data without a normal distribution. Categorical data were presented as case numbers (percentages), and comparisons between groups were made using the chi-square test. Multivariate logistic regression analysis was utilized to assess the association between various factors and abnormal HC (macrocephaly and microcephaly), and the results as odds ratios (ORs) with 95% confidence intervals (CIs). The statistical analyses were conducted using SPSS 22.0, and a two-sided *P*-value of <.05 was considered statistically significant for between-group comparisons.

## 3. Result

**Figure 1.**
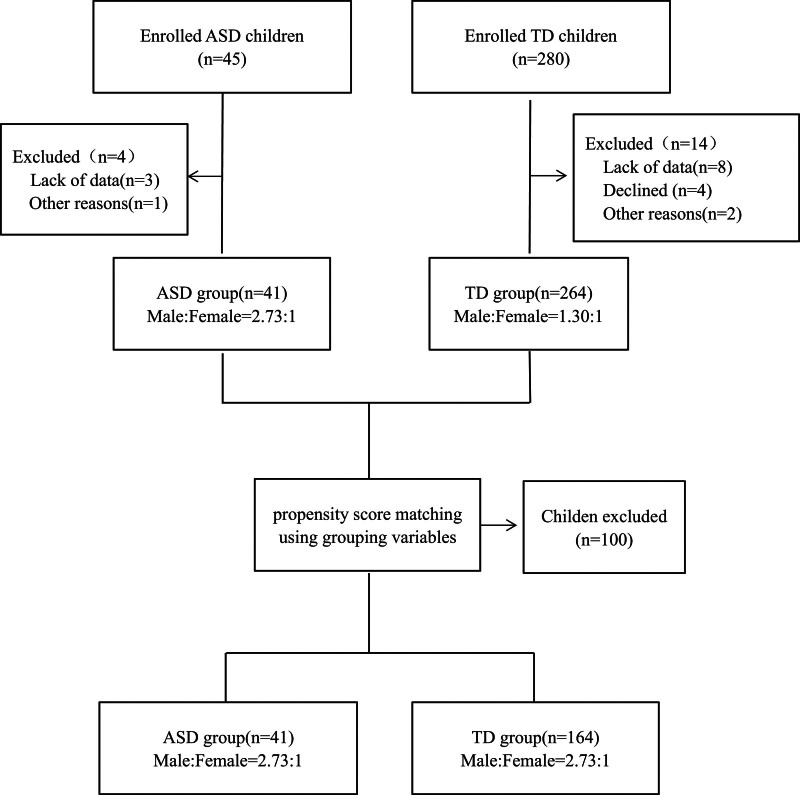
Flowchart of the study design. ASD = autism spectrum disorder, TD = typical development.

Between March 2018 and February 2019, a total of 305 children were enrolled in this study. Among them, 41 children had ASD and 264 children had TD. After PSM, the final analysis included 41 children with ASD and 164 children with TD. The study design and flowchart are depicted in Figure 1.

### 3.1. Gender and age characteristics of research subjects before and after PSM matching

A total of 305 Han children participated in this study, consisting of 41 children with ASD and 264 children with TD. Table 1 displays the gender and age characteristics of the participants. The ASD group had a gender ratio of approximately 2.73:1, while the TD group had a ratio of 1.30:1. The difference in gender distribution between the 2 groups was statistically significant (*P* < .05). However, there was no significant difference in age between the ASD and TD groups (*P* > .05).

**Table 1:** Characteristics of children before PSM matching.

variable	TD group (N = 264) n (constituent ratio %)	ASD group (N = 41) n (constituent ratio %)	χ^2^/Z	*P*
Gender			4.10	.04
Male	149 (56.4)	30 (73.2)		
Female	115 (43.6)	11 (26.8)		
Age (years)	5 (4, 6)	5 (4, 6)	0.06	.95

ASD = autism spectrum disorder, TD = typical development.

After applying PSM for gender and age, the characteristics of the 2 groups were well balanced (Table 2).

**Table 2:** Characteristics of children after PSM matching.

Variable	TD group (N = 164) n (constituent ratio%)	ASD group (N = 41) n (constituent ratio%)	*χ*^2^/*Z*	*P*
Gender			<0.01	>.99
Male	120 (73.2)	30 (73.2)		
Female	44 (26.8)	11 (26.8)		
Age (years)	5 (3, 6)	5 (4, 6)	0.68	.49

ASD = autism spectrum disorder, TD = typical development.

### 3.2. General demographic characteristics of the 2 groups of subjects

The demographic characteristics of the children in the ASD and TD groups were analyzed separately. Statistical analysis revealed no significant differences between the 2 groups (Table 3).

**Table 3:** General demographic characteristics.

	ASD group (N = 41)n (constituent ratio %)	TD group (N = 164) n (constituent ratio %)	*Z*/χ^*2*^	*P*
Maternal age (years)	28 (27, 30)	28 (26, 30)	0.59	.55
Paternal age (years)	29 (27, 33)	29 (27, 31)	0.80	.42
Maternal education level			0.78	.38
High school and below	7 (17.1)	24 (14.6)		
Junior college education	18 (43.9)	65 (39.6)		
Undergraduate course	14 (34.1)	60 (36.6)		
Master’s degree or above	2 (4.9)	15 (9.2)		
Paternal education level			1.05	.31
High school and below	12 (29.3)	36 (22.0)		
Junior college education	15 (36.6)	64 (39.0)		
Undergraduate course	13 (31.7)	52 (31.7)		
Master’s degree or above	1 (2.4)	12 (7.3)		
Maternal previous history of autoimmune diseases ^*^			<0.01	>.99
Yes	1 (2.4)	2 (1.2)		
No	40 (97.6)	162 (98.8)		
Paternal previous history of autoimmune diseases^*^			0.03	.86
Yes	1 (2.4)	1 (0.6)		
No	40 (97.6)	163 (99.4)		

ASD = autism spectrum disorder, TD = typical development.

*Previous history of autoimmune diseases refers to the presence or absence of Hashimoto's thyroiditis, ankylosing spondylitis, rheumatoid arthritis, and other diseases before pregnancy.

### 3.3. Anthropometric characteristics of the 2 groups of subjects

Based on the developmental indicators of HC in children from 9 cities in China in 2005, the ASD group had an incidence rate of 22.0% (n = 9) for macrocephaly (HC greater than 2 standard deviations for the same age and gender) and 17.1% (n = 7) for microcephaly (HC less than 2 standard deviations for the same age and gender). In the TD group, the incidence of macrocephaly was 1.8% (n = 3), and the incidence of microcephaly was also 1.8% (n = 3).

Anthropometric measurements were analyzed for children in the ASD and TD groups. The findings revealed that children in the ASD group had higher weight and a higher occurrence of HC abnormalities, specifically macrocephaly and microcephaly, compared to the TD group. These differences were statistically significant (*P* < .05) as shown in Table 4.

**Table 4:** Median height, weight (interquartile range) and abnormal cases of head circumference and outer ear (percentage).

	ASD group	TD group	*Z*/χ^*2*^	*P*
Height	115 (104.1, 121)	109.85 (101.4, 119.98)	1.65	.10
Weight	21.6 (18, 27.85)	18.93 (16.35, 23.05)	3.25	<.01
Macrocephaly	9 (22.0%)	3 (1.8%)	20.59	<.01
Microcephaly	7 (17.1%)	3 (1.8%)	13.3	<.01

ASD = autism spectrum disorder, TD = typical development.

### 3.4. Analysis of risk factors in ASD group

Logistic regression analysis was conducted to examine factors related to mothers’ pregnancy and childbirth in the ASD and TD groups. Table 5 presents the results of the analysis. A statistically significant association was observed between children with ASD and maternal FA supplementation during pregnancy (*P* < .05). The OR with a 95% CI was calculated as 3.69 (1.76, 7.76), indicating that the lack of FA supplementation during pregnancy posed a risk factor for ASD. However, no statistically significant association was found between premature birth and history of complications during pregnancy (*P* ≥ .05).

**Table 5:** Logistic regression analysis of factors related to children’s ASD and maternal pregnancy.

Factor	ASD group n (%)	TD group n (%)	χ^2^	OR (95%CI)	*P*
Premature birth			0.23	0.7 (0.16, 3.00)	.63
Yes	3 (7.32)	12 (7.32)			
No	38 (92.68)	152 (92.68)			
Maternal history of complications during pregnancy					
Infection	16 (39.02)	57 (34.76)	0.09	0.89 (0.41, 1.93)	.76
Anemia	12 (29.27)	65 (39.63)	2.34	1.84 (0.84, 4.04)	.13
Abnormal thyroid function	2 (4.88)	15 (9.15)	0.95	2.25 (0.44, 11.44)	.33
Hypertension	1 (0.00)	12 (7.31)	0.46	2.11 (0.24, 18.27)	.50
Hyperglycaemia	3 (7.32)	26 (15.85)	2.19	2.64 (0.73, 9.56)	.14
Maternal folic acid supplementation during pregnancy			11.86	3.69 (1.76, 7.76)	<.01
Yes	16 (39.02)	116 (70.73)			
No	25 (60.98)	48 (29.27)			

ASD = autism spectrum disorder, CI = confidence interval, OR = odds ratio, TD = typical development.

### 3.5. Effects of maternal pregnancy-related factors on HC

The objective of this study was to further investigate the influence of maternal factors during pregnancy and childbirth on HC. It was observed that risk factors without abnormal signs, such as macrocephaly, were not present in premature infants or in mothers with abnormal thyroid function or hypertension during pregnancy. Additionally, microcephaly did not occur in premature infants or in mothers with abnormal thyroid function, hypertension, or hyperglycemia during pregnancy. Logistic regression analysis was conducted to examine factors associated with abnormal HC.

The analysis of risk factors for macrocephaly revealed a statistically significant association between macrocephaly and maternal FA supplementation before pregnancy (*P* < .05). The OR with a 95% CI was calculated as 4.16 (1.18, 14.60), indicating that mothers who did not supplement FA during pregnancy were at risk for macrocephaly. The analysis also explored the risk factors for deformities and these are presented in Table 6.

**Table 6:** Logistic regression analysis of factors related to macrocephaly and maternal pregnancy.

Factor	Macrocephaly (N = 12)n (%)	Control group (N = 193)n (%)	χ^*2*^	OR (95%CI)	*P*
Maternal history of complications during pregnancy					
Infection	4 (33.33)	69 (35.75)	0.29	1.42 (0.40, 5.10)	.59
Anemia	2 (16.67)	75 (38.86)	1.05	2.04 (0.52, 7.97)	.31
Hyperglycaemia	1 (8.33)	28 (14.51)	0.37	1.93 (0.23, 15.92)	.54
Maternal folic acid supplementation during pregnancy			4.95	4.16 (1.18, 14.60)	<.01
Yes	4 (33.33)	128 (66.32)			
No	8 (66.67)	65 (33.68)			

CI = confidence interval, OR = odds ratio.

On the other hand, the analysis of factors related to microcephaly did not yield statistically significant results, as shown in Table 7.

**Table 7:** Logistic regression analysis of factors related to microcephaly and maternal pregnancy.

Factor	Microcephaly (N = 10)n (%)	Control group (N = 195)n (%)	χ^*2*^	OR (95%CI)	*P*
Maternal history of complications during pregnancy					
Infection	5 (50.00)	68 (34.87)	0.51	0.62 (0.17, 2.28)	.47
Anemia	3 (30.00)	74 (37.95)	0.22	1.40 (0.35, 5.66)	.64
Maternal folic acid supplementation during pregnancy			2.20	2.70 (0.73, 10.05)	.14
Yes	4 (40.00)	128 (65.64)			
No	6 (60.00)	67 (34.36)			

CI = confidence interval, OR = odds ratio.

## 4. Discussion

ASD is a complex neurodevelopmental condition that has a significant impact on individuals, families, and society. The clinical and etiological diversity of ASD presents challenges in identifying early biomarkers. However, there are several compelling reasons to continuing to strive for an early detection and diagnosis. Firstly, there is a considerable delay between the initial identification of a problem by the parents and the diagnosis of ASD. Secondly, early diagnosis provides an opportunity for children with ASD to benefit from early intervention, which can improve outcomes for both the child and community. Finally, understanding the underlying pathogenic mechanisms is essential in comprehending the biological phenotype of ASD and has broader scientific significance.

This study found that 22.0% of children with ASD had macrocephaly and microcephaly, and the incidence of microcephaly was significantly higher at 17.1%, compared to the TD group, where the incidence of both macrocephaly and microcephaly was only 1.8%.

An abnormal HC indicates atypical brain size, which is determined by a complex series of coordinated processes involving neural stem cell proliferation, expansion, and migration. Extensive research conducted on children with cortical dysplasia demonstrates that brain size is a significant predictor of functional impairment.^[20]^ The expansion of the human brain, particularly the neocortex, is a remarkable evolutionary process associated with cognition, emotion, and social skills. Any disruption in the steps of cortical expansion can result in abnormalities such as microcephaly or macrocephaly.^[21]^ Rahaman et al^[22]^ identified a trend toward increased HC in Bangladeshi children with ASD, while men with ASD exhibited significantly larger HC than men without ASD. Individuals with larger HC tended to have more severe ASD core characteristics. Bedford et al^[23]^ observed a significant increase in the cortical volume, average cortical thickness, and total brain volume in an ASD population. Vertex analysis revealed greater cortical thickness in specific areas such as the inferior frontal and prefrontal cortices, superior temporal, posterior central, posterior cingulate gyrus, and anterior cuneiform lobe in the ASD group compared to the control group. A study by Ben-Itzchak et al^[24]^ reported a higher incidence of microcephaly in individuals with ASD (5.9%), compared to the general population (3%). Notably, the prevalence of microcephaly was significantly higher in women (15.1%) than that in men (4.5%).

The study findings indicate that the absence of maternal FA supplementation during pregnancy is a risk factor for both ASD and macrocephaly. However, there was no observed impact of premature delivery or pregnancy complications (such as respiratory tract infection, anemia, thyroid dysfunction, hypertension, and hyperglycemia) on the occurrence of ASD or abnormalities in HC.

Folate plays a crucial role in regulating of nucleotide synthesis and methylation.^[25]^ Insufficient dietary folate leads to elevated levels of homocysteine in the serum, which in turn causes oxidative stress, DNA fragmentation, and apoptosis.^[26]^ Various studies have demonstrated that high Hcy levels during pregnancy can have teratogenic effects. Folic acid (FA) is known to exert a protective effect by reducing serum homocysteine levels through the diversion of excess homocysteine into the methionine metabolic pathways.^[27]^

Maternal lack of FA supplementation during pregnancy has been identified as a risk factor for ASD, which is consistent with previous studies. Braun et al^[28]^ indicated that prenatal multivitamin/FA supplementation may decrease the risk of ASD in a Canadian population. Levine et al^[29]^ discovered that maternal exposure to FA before and during pregnancy reduced the risk of ASD in offspring compared with those whose mothers were not exposed to it. However, conflicting findings have been reported by other studies. Virk et al^[12]^ and Strøm et al^[11]^ did not find that the use of FA supplements in early pregnancy reduces the risk of ASD in offspring. Interestingly, high folate levels at birth were associated with an increased risk of ASD in children in the Boston Birth Cohort Study.^[30]^ Paul et al^[31]^ observed an association between elevated circulating folate levels and ASD risk, possibly due to changes in folate-dependent carbon metabolism enzyme activity and/or impaired CBL function. This suggests that the increase in total FA serum levels may be an epiphenomenon of ASD pathogenesis, rather than a causative factor.^[32]^ Another possible explanation could be the impaired absorption and utilization of FA from the blood into cells. One study revealed a common biomarker in children with ASD and their parents: the presence of serum autoantibodies against folate receptor α, leading to impaired physiological FA transfer across the blood–brain and placental barriers to the brain and fetus.^[33]^ The conversion of FA to 5,10-methylenetetrahydrofolate, a process facilitated by methylenetetrahydrofolate reductase, is necessary for FA utilization. A meta-analysis demonstrated that the methylenetetrahydrofolate reductase C677T polymorphism is a risk factor for ASD.^[34]^

A limited number of studies have explored the correlation between maternal failure to supplement FA during pregnancy and abnormalities in HC. Rehman et al^[35]^ found that maternal FA supplementation during pregnancy resulted in increased HC as a result of hydrocephalus. In a population-based prospective cohort study conducted in Rotterdam, the Netherlands, Zou et al^[36]^ discovered that maternal FA deficiency during pregnancy was associated with smaller total brain volume and white matter. On the other hand, Petry et al^[37]^ revealed that FA supplementation during pregnancy had no impact on HC. The effect of FA on HC may be linked to its influence on the neural crest. Melo et al^[38]^ demonstrated that elevated levels of homocysteine in vitro can enhance the growth and proliferation of neural crest cells in the mice head, potentially leading to abnormal development of neural crest-related diseases in animal embryos. These findings suggest that folate deficiency is associated with aberrations in the growth, differentiation, and migration of neural crest cells, which may provide an explanation for folate deficiency being identified as a risk factor for both ASD and abnormal HC in this study.

Our study found no statistically significant correlation between Autism Spectrum Disorder (ASD) and several factors that have previously been associated with the occurrence of ASD in offspring. These factors include maternal age at delivery, educational background, parental history of autoimmune diseases, gestational age, delivery method, and pregnancy complications such as infection, anemia, thyroid dysfunction, hypertension, and hyperglycemia. Prior research has reported a correlation between maternal age at delivery,^[39]^ educational background,^[40]^ parental history of autoimmune diseases,^[41,42]^ gestational age,^[39,43]^ delivery method,^[44]^ pregnancy complications, ^[43]^ and ASD in offspring, while some studies contradict these findings.^[45–47]^ However, these disparate results might stem from the small number of cases analyzed in some investigations and differences in racial backgrounds. As a result, further large-scale research is necessary to validate these conclusions.

Although our study provides important insights into the incidence rate of HC abnormalities in children with ASD, it is important to acknowledge its limitations. First, our investigation only assessed the incidence rate of HC abnormality in children with ASD and did not determine whether it existed at birth or was a consequence of later growth and development. Therefore, further cohort studies need to be conducted to establish the timing of this condition’s onset. Secondly, this research was based on a small sample size and conducted at a single center. Despite utilizing PSM to match more TD groups, the number of participants in the ASD group was still relatively low. Thus, future large-scale and multicenter studies are necessary to overcome these limitations and expand upon our findings.

## 5. Conclusion

The incidence of abnormal HC was found to be higher in the ASD group than in the TD group. Interestingly, maternal FA supplementation during pregnancy appears to have a potential negative association with the occurrence of ASD and abnormal HC in offspring. These findings highlight the importance of conducting further investigations into the role of maternal health status in the etiology of ASD. Therefore, it is recommended that additional studies be undertaken to explore this relationship in greater detail.

## Author contributions

**Resources:** Shichun Zhao.

**Writing – original draft:** Shichun Zhao.

**Writing – review & editing:** Lei Liu.
